# Nasal Obstruction and Catarrh*Read before the Augusta Academy of Medicine, December 5, 1894.

**Published:** 1895-03

**Authors:** Austin S. Tinsley

**Affiliations:** Augusta, Ga.; Member of the Association of Military Surgeons of the United States; Former Demonstrator of Anatomy Medical Department University of Georgia, etc.


### NASAL OBSTRUCTION AND CATARRH.*

By AUSTIN S. TINSLEY, M.D., AUGUSTA, GA.

Member of the Association of Military Surgeons of the United States; Former Demonstrator of Anatomy Medical Department University of Georgia, etc.

It is stated by rhinologists of extended observation, that ninety per cent. of the American people suffer to a greater or less extent from some form of chronic nasal or naso-pharyngeal catarrh.

A subject which involves nine out of every ten persons with whom we come in contact engages, at once, our careful and thoughtful attention. Nasal passages which are sufficiently free to admit of the unobstructed entrance and exit of air may be safely said to be a necessity to the enjoyment of perfect health and immunity from catarrhal and resultant sequel affections; as well as a prerequisite to the successful treatment of any of the forms of chronic rhinitis.

Extensive obstruction to nasal respiration is the direct cause of mouth breathing. At each inspiratory act the air beyond the point of obstruction becomes rarefied; the mucous membrane forming the walls of this partial vacuum becomes constantly, passively congested; and in young subjects the yielding bony framework gives to the continued atmospheric pressure exerted on the exterior, and the superior maxilla contracts, the hard palate becomes abnormally arched, and the lumen of the nares unnaturally small. The "nasal twang," or "talking through the nose," is due to the absence of the reinforcement of the voice by normal nasal resonance, and can always be relieved by the establishment of free nasal respiration.

Acute nasal catarrh is most usually the result of exposure of the body to cold while overheated, or the converse, exposure of the body when cold, too suddenly to an overheated atmosphere.

---

*Read before the Augusta Academy of Medicine, December 5, 1894.

Repeated attacks of acute rhinitis gradually reduce to a chronic form the over-distended paretic condition of the vascular supply inducing the formation of new and pathological elements, thus encouraging the development of structural changes of the mucous membrane, and a change in the character of the mucus secreted.

Thickening of the epithelial layer of the mucous membrane, as well as the deeper cellular structure, results in an obstructive hypertrophic condition of the turbinated bodies.

Occlusion of the respiratory passages of the nose, partial or complete, may be produced by deflections of the nasal septum, bony ridges along the line of the suture of the septum, with the superior maxillary bone, morbid growths, ecchondromata, osteoma, and exostoses.

I am convinced from a careful study of the subject and from considerable clinical observation, that nasal obstruction is the etiological cause of a very large per cent. of chronic catarrh of the nose and throat; and when obstruction occurs as a complication of catarrhal conditions, which may have their origin in a scrofulous, specific, or tubercular diathesis, on the removal of such obstruction will depend the highest degree of success attainable from the therapeutic agencies employed.

To nasal obstruction may be attributed, indirectly, a considerable proportion of the ear and throat troubles which come under our observation.

The physiological function of the nose in supplying heat and moisture to the inspired air not being performed, cold and dry air, with its dust and irritating substances, reaches the throat, larynx, and lungs through the process of mouth breathing. Follicular pharyngitis, pharyngitis sicca, and catarrhal laryngitis supervene; and in persons predisposed, phthisis may develop.

R. E. Swinburne, of New York, made a careful study of 1,000 cases of otitis media, and found as a result of his observations that 950 cases presented some pathological condition of the nasal and naso-pharyngeal mucous membrane.

Chronic congestion of the nasal mucous membrane and mechanical obstruction of the nares may have a decided reflex influence in the development of other diseases.

Bosworth, of New York, found that in eighty-eight cases of asthma which came under his treatment, there were in the great majority of instances nasal polypi, deflected septi, or hypertrophic rhinitis.

H. L. Swain, of New Haven, regards the treatment of intra-nasal abnormalities as of as much or more value in the management and cure of asthma as all other methods combined.

Kibbe, of Seattle, reports a case of persistent asthenopia which yielded promptly to the removal of a large hypertrophy of the middle turbinated bone.

Bates, of New York, reports a case of myopia in which vision was greatly improved by the removal of a cartilaginous growth on the septum.

Constant headaches due to congestion of the frontal sinuses are promptly cured by the relief of nasal obstruction and catarrhal conditions.

Nasal obstructions differ very widely in their nature and extent, and even more widely in the degree of inconvenience the same amount of obstruction produces in different individuals.

In children under eight years old adenoid growths of the vault of the pharynx and hypertrophy of the nasal mucous membrane are the most common cause of obstruction. Deflections at so early an age are very rare. Children having such a form of obstruction are invariably strumous, and repeated colds or an attack of an infectious disease will act as an exciting cause.

Owing to the inequality and rapidity of the development of the septum after the seventh year, deflections are particularly liable to occur, producing an abnormal relation between the bony walls of the nostrils which so encroach upon their caliber as to prevent the proper expansion of the mucous membrane incident to the sudden changes of the surrounding temperature.

In addition to the more radical deflection of the bony structure, deformities of variable degree and variety of the triangular cartilage are often to be found, and in nearly every instance they are due to traumatism.

The clinical observation of ophthalmologists in the treatment of granulated lids and refractory diseases of the conjunctiva, daily demonstrate that the relief of an obstructive nasal catarrh is followed by a prompt response to the ocular treatment.

Obstruction of the nose affects the ear pathologically in two ways: First, when we inhale or swallow, the air in the space beyond the point of obstruction becomes partially exhausted, at the same time the air in the middle ear becomes rarefied by means of the Eustachian tube; the equilibrium of the drum membrane is disturbed by the greater atmospheric pressure in the external meatus, the drum is depressed, the lining membrane of the tympanic cavity, under the influence of the partial vacuum, becomes continuously congested, which may result in an incurable catarrhal disease of the middle ear.

The second and less frequent way is through direct extension by continuity of structure of the inflammatory catarrhal process to the middle ear.

The nature and extent of nasal obstructions are so various and the expedients required in each case so different, that no one method of operation can be adopted which will suit all cases, and occasionally the best plan to be pursued will tax the resources and skill of the most experienced operator.

